# Variation of Bacterial and Archaeal Community Structures in a Full-Scale Constructed Wetlands for Wastewater Treatment

**DOI:** 10.1155/2018/9319345

**Published:** 2018-10-16

**Authors:** Xiu-lu Lang, Xiang Chen, Ai-ling Xu, Zhi-wen Song, Xin Wang, He-bing Wang

**Affiliations:** School of Environmental and Municipal Engineering, Qingdao University of Technology, Qingdao 266033, China

## Abstract

Microorganisms play important roles in the reduction of organic and inorganic pollutants in constructed wetlands used for the treatment of wastewater. However, the diversity and structure of microbial community in constructed wetland system remain poorly known. In this study, the Illumina MiSeq Sequencing of 16S rDNA was used to analyze the bacterial and archaeal microbial community structures of soil and water in a free surface flow constructed wetland, and the differences of bacterial communities and archaeal compositions between soil and water were compared. The results showed that the Proteobacteria were the dominant bacteria, making up 35.38%~48.66% relative abundance. Euryarchaeotic were the absolute dominant archaea in the influent sample with the relative abundance of 93.29%, while Thaumarchaeota showed dominance in the other three samples, making up 50.58%~75.70%. The relative abundances of different species showed great changes in bacteria and archaea, and the number of dominant species in bacteria was much higher than that in archaea. Compared to archaea, the community compositions of bacteria were more abundant and the changes were more significant. Meanwhile, bacteria and archaea had large differences in compositions between water and soil. The microbial richness in water was significantly higher than that in soil. Simultaneously, soil had a significant enrichment effect on some microbial flora.

## 1. Introduction

As a new type of sewage treatment system, constructed wetlands have gradually entered the field of vision. Constructed wetlands for wastewater treatment were widely used in developed countries, such as the United States and Germany, because of its low costs, good removal rates for organic substances and also for nutrients (N, P), and higher surface water quality [1]. Shandong Province had built many constructed wetlands which occupied 7.6% of the land [2] and mainly distributed in Nansi Lake and Dongping Lake [3]. The constructed wetlands could remove pollutants through providing habitats for microbes to stimulate their activities [4]; therefore, microorganisms were particularly important in the reduction of organic and inorganic pollutants in constructed wetlands. Due to the uncertainty and variability of the distribution of microbial community structure in constructed wetlands, it had aroused the interest and attention of scholars.

At present, extensive researches have been conducted on microbial community structure of sewage treatment systems [5–7]. Recently, with the development of high-throughput sequencing technology, it has also been widely used in environmental samples, such as the bacterial community structures in airborne [8] and water [9] and the archaeal community structures in soil [10], even in the sludge of wastewater treatment [11]. However, the above studies have rarely analyzed the bacterial and archaeal community structures of the same samples at the same time. Similar studies also show significant differences due to environmental differences in the study sites.

Therefore, in this study, the water and soil samples, collected from a free surface flow constructed wetland, were assessed by Illumina MiSeq high-throughput method, the objective was to investigate the microbial community structures and compare the microbial abundance differences between water and soil, including bacteria and archaea.

## 2. Methods

### 2.1. Sampling Sites

**Figure 1:**
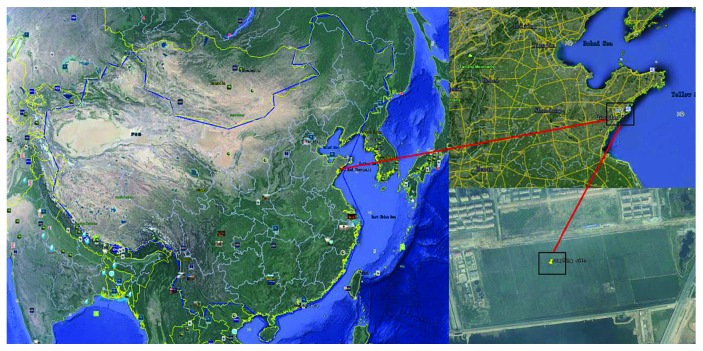
Map showing the location of the sampling sites in constructed wetland.

The free water surface constructed wetland, located in the interior of Huangdao District (Qingdao City, Shandong Province, China), at a latitude of 35°35′ to 36°08′ north and a longitude of 119°30′ to 120°11′ east, is a part of an integrated sewage purification system. This region has a warm temperate continental monsoon with a mean annual temperature of 12.0°C and a mean annual precipitation of 794 mm. The constructed wetland wastewater treatment system had a total area of 76.7 hm^2^ and a treatment capability of 3.0 × 10^4^ m^3^·d^−1^ and was surrounded by the Yellow Sea on east and south. It consisted of 99 treatment beds and received secondary unchlorinated wastewater from Jiaonan Municipal Wastewater Treatment Facility with A^2^O as the secondary treatment. All beds were planted with common reed (*Phragmites australis*) and a number of naturally germinated wetland plants (*Typha orientalis*, *Scirpus validus*, *Lemna minor*, etc.). To facilitate the harvest progress of above-ground biomass, sewage did not enter the constructed wetland bed from December to March of next year. In this study, two different constructed wetland treatment units with and without sewage water were selected, wet soil and dry soil from each unit, and influent and effluent from unit with sewage water were sampled in May 2017. Detailed geographic information of the sampling sites is shown in Figure 1.

### 2.2. Sampling Methods

50 g of soil sample and 10 L of water sample were collected from each sample site by sterile sealed bags and sterile bottles, respectively. After removing the fine roots in soil samples, the water and soil samples were transferred to the laboratory immediately. After dewatered by centrifugation, a fraction of the soil samples were stored at −20°C for molecular analysis. A part of water samples was filtered by a vacuum pump with 45-mm-diameter microporous membrane, then using douching and centrifugation method carefully transferred into 2 mL sterile centrifuge tubes and stored at −20°C until DNA extraction; meanwhile, the other part was stored at 4°C for chemical analysis.

### 2.3. DNA Extraction

Soil DNA and water DNA were extracted from 500 mg of frozen soil and 500 mg of filter residue, respectively, using a Soil DNA Kit (OMEGA, China) according to the manufacturer's instructions. The extracted DNA was checked using the UV/nucleic acid protein detector (IMPLEN, Germany).

### 2.4. Illumina MiSeq Sequencing

Targeting target sequences reflects the compositions and diversities of microbes, designing corresponding primers according to the conserved regions in the sequences and adding sample-specific barcode sequences to further amplify the variable region of the rRNA gene (single or continuous) or specific gene fragments for PCR amplification. PCR amplification products were detected by 2% agarose gel electrophoresis, and the target fragment was excised from the gel. PCR products were recovered for fluorescence quantification, according to the needs of each sample sequencing volume, and the samples were mixed in the appropriate ratio. Sequencing libraries were prepared using Illumina's TruSeq Nano DNA LT Library Prep Kit and on the machine for high-throughput sequencing.

### 2.5. Sequence Data Analyses

In order to integrate the original double-end sequencing data, the two-terminal sequence of FASTQ format was first screened by sliding window. The size of the window is 10 bp and the step size is 1 bp. Starting from the first base position on the 5′ end, the average base mass in the window is ≥Q20 (i.e., the base average measurement accuracy is ≥99%). From the first truncated sequences at windows, with average mass values below Q20, we requir a truncated sequence ≥150 bp in length with no ambiguous base N allowed. Subsequently, the FLASH software [12](v1.2.7, http://ccb.jhu.edu/software/FLASH/) was used to pair the double-stranded sequences that passed the quality screening according to overlapping bases. It is required that the overlapping base length of two sequences of read 1 and read 2 be ≥10 bp and the base mismatch is not allowed. Finally, based on the index information (i.e., barcode sequence, for the beginning of the sequence used to identify a small base sequence) corresponding to each sample, the connected sequence identification is assigned to the corresponding sample (requires index sequence exactly match), to obtain a valid sequence for each sample.

## 3. Results

### 3.1. Physical and Chemical Characteristics of Soil and Water in Constructed Wetlands

The results of soil and water basic properties were listed in Table 1. All the chemical indicators of wet soil were far below the dry soil, especially content of organic matter, and dry soil was about 15 times more than wet soil. The constructed wetlands had a very good purification effect; ammonia nitrogen and nitrite nitrogen in effluent decreased obviously. Simultaneously, the content of dissolved oxygen also improved.

### 3.2. Bacterial Community Structures of Soil and Water in Constructed Wetlands

#### 3.2.1. Bacterial Alpha Diversity Analysis

**Figure 2:**
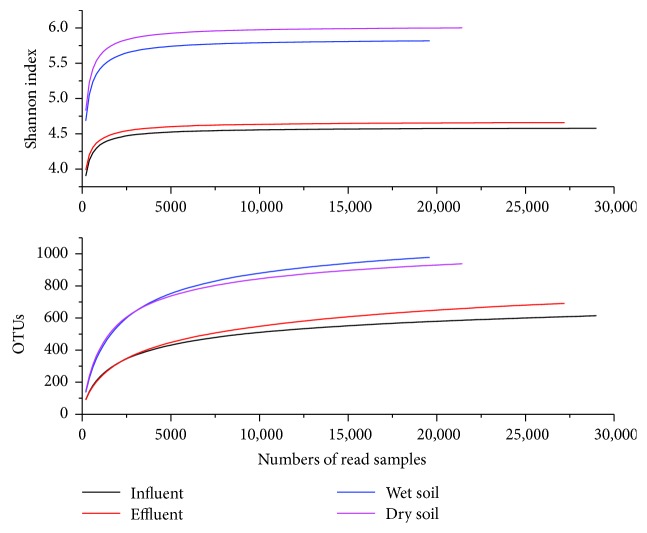
Bacterial rarefaction curves and Shannon diversity index curves.

Rarefaction curves of the four samples were shown in Figure 2. The rarefaction curves and Shannon diversity index curves clearly revealed that the bacterial community structures of soil samples were considerably higher than those in water samples. Two kinds of curves tended to be gentle, suggesting that the sequencing results had been enough to reflect the diversity of the current sample, and increasing the depth of sequencing could not detect more new OUTs. The sequencing results could basically reflect the microbial community structures of four samples.

A total of 29,000, 27,204, 19,597, and 21,439 trimmed reads for samples influent, effluent, wet soil, and dry soil were obtained, respectively, after the removal of unqualified reads (Table 2). ACE estimator [13] and Chao1 estimator [14] were used to estimate the number of species actually present in the community. The greater Chao1 estimator, the higher richness of the community, and so was the ACE estimator. The community richness in soil samples was much higher than that in water samples, showing microbes were more likely to attach to solid particles. Shannon diversity index [15] and Simpson index [16] were both the commonly used index for evaluating community diversity; the higher Shannon index and the lower Simpson diversity index could explain the higher community diversity. The community diversity in dry soil sample was the highest in this study, while that in influent sample was the lowest. Simpson index was more sensitive to uniformity and dominant OTUs in the community, and it demonstrated a high degree of uniformity in four samples.

#### 3.2.2. Bacterial Community Structures of Soil and Water in Constructed Wetlands

**Figure 3:**
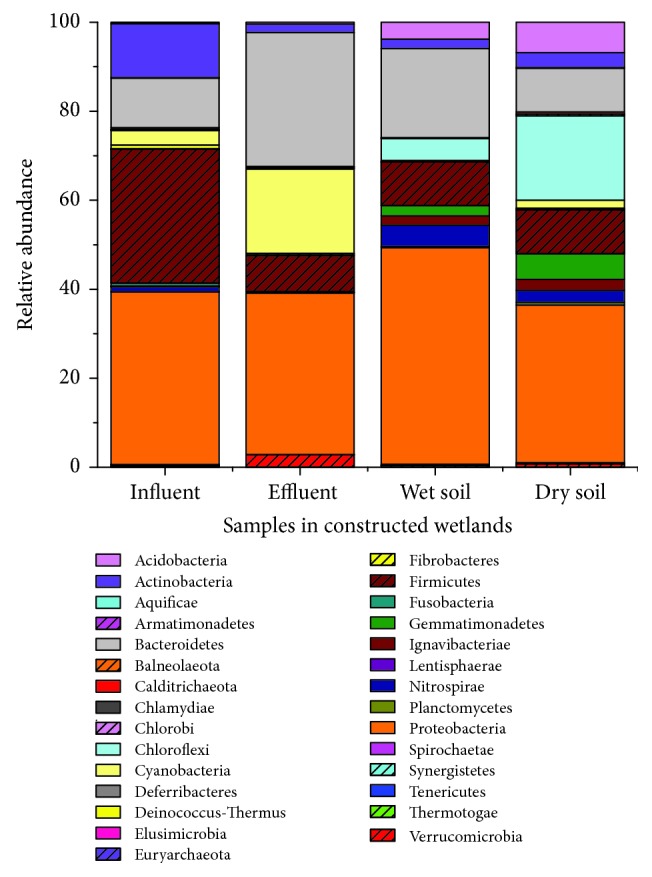
Bacterial relative abundance of four samples in phyla in constructed wetlands.

Bacterial sequences in the four samples were classified into taxonomic classes using the default settings of the mothur platform. A total of 29 bacterial phyla were found in this study. The total phylum numbers in influent, effluent, wet soil, and dry soil were 22, 23, 23, and 23, respectively. Four samples were similar in the number of phyla levels, but quite different in compositions, and the detailed relative abundances were shown in Figure 3. In the four samples, Proteobacteria, Firmicutes, Actinobacteria, Bacteroidetes, Cyanobacteria, and Chloroflexi were the most common bacterial phyla with a high relative abundance, while the proportion of the other phyla were very low. Proteobacteria were the most dominant phylum in the four samples with the relative abundance of 35.38%~48.66%. Firmicutes in influent sample (30.12%) and Bacteroides in effluent (30.03%) and wet soil (20.05%) samples also showed in absolute superiority. Different from the other three samples, the proportion of Chloroflexi in dry soil was high, accounting for 18.96%. The community structures of the two water samples were more similar, and so were the two soil samples. Most bacterial phyla were found in all 4 samples, such as Verrucomicrobia, Planctomycetes, and Ignavibacteriae. However, Aquificae, Lentisphaerae, and Synergistetes were emerged only in the water samples, while Thermotogae, Deferribacteres, Calditrichaeota, and Armatimonadetes existed only in the soil samples. Tenericutes and Fusobacteria were detected in all samples except dry soil, while Balneolaeota were emerged only in the effluent with a very low relative abundance. It is worth noting that Euryarchaeota, which belonged to archaea, were also detected in this bacterial sequencing.

**Figure 4:**
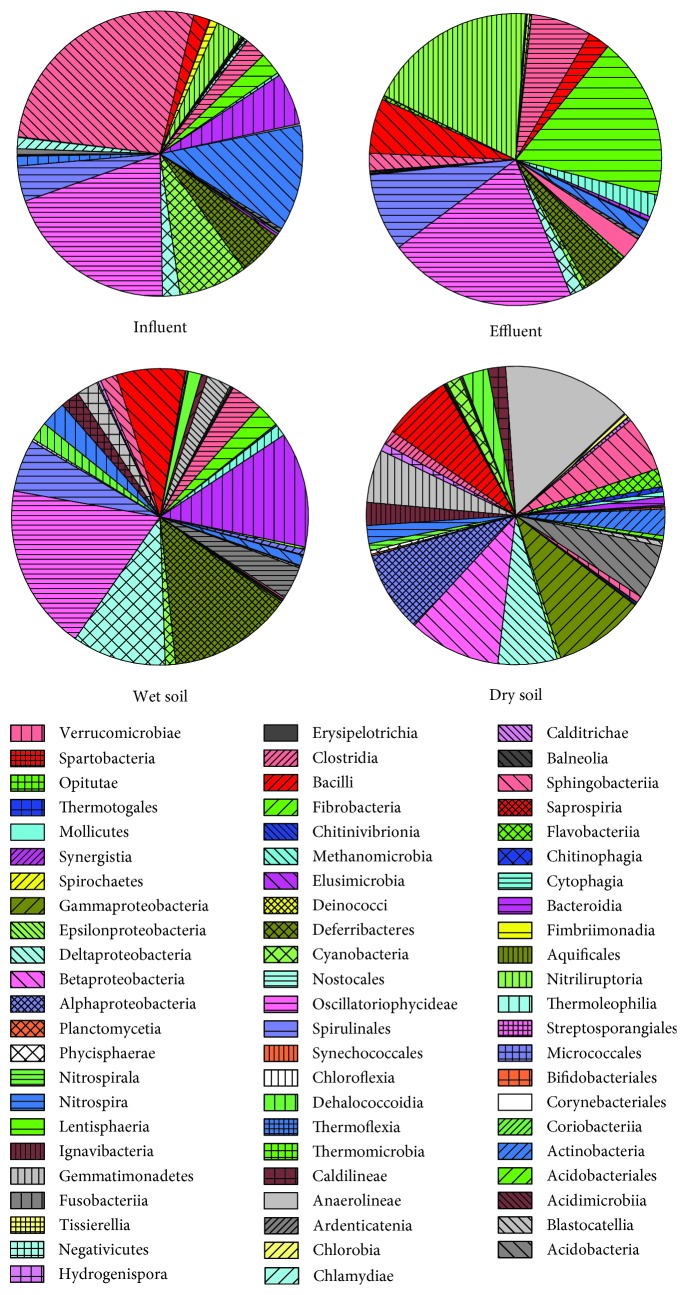
Bacterial relative abundance of four samples in classes in constructed wetlands.

The distribution characteristics of classes were analyzed, and the results were shown in Figure 4. A total of 68 bacterial classes were found in this study. The total class numbers in influent, effluent, wet soil, and dry soil were 44, 47, 55, and 53, respectively. The abundance distributions of Alphaproteobacteria, Sphingobacteriia, and Gammaproteobacteria in the four samples were relative average, while the relative abundances of the other classes were quite different. Clostridia, Actinobacteria, and Epsilonproteobacteria were the dominant classes in the influent with the relative abundance of 26.91%, 11.96%, and 7.76%, respectively, while they did not exceed 3% in the other three samples; however, the relative abundances of Fusobacteriia were obviously higher in influent than the other three samples. The relative abundances of Fimbriimonadia and Fibrobacteria in wet soil and Ignavibacteria in dry soil were much higher than the other three samples. Cyanobacteria and Flavobacteriia were most frequently detected in effluent accounting for 18.84% and 18.02%, respectively, while they showed a lower relative abundance in the other three samples. The relative abundances of Betaproteobacteria were less than 10% in the dry soil, while it showed advantage in the other three samples accounting for 18.15%~20.9%. Coriobacteriia, Chloroflexia, etc., a total of 19 classes, were only detected in the soil samples, and among them, there were 3 classes only in wet soil and 5 classes in dry soil. Deltaproteobacteria, Erysipelotrichia, etc., a total of 8 classes, were only detected in the water samples and 3 classes emerged only in effluent.

Due to the huge amount of data, the dominant genera, with relative abundances over 1%, were listed in Table 3. A total of 40 bacterial genera were found. The total genus numbers in influent, effluent, wet soil, and dry soil were 28, 33, 28, and 22, respectively. In influent sample, in addition to *Mycobacterium* and *Rhodoferax*, the other 10 dominant genera in the other three samples, the relative abundances were all less than 1%. *Haliscomenobacter*, *Synechococcus*, *Polaribacter*, and *Owenweeksia* were emerged only in the effluent, simultaneously, and *Herminiimonas*, *Prevotella*, *Vogesella*, *Trichococcus*, and *Dysgonomonas* were detected only in the water samples. The quantities of dominant genera in the soil samples were lower than those of the water samples, obviously. *Tangfeifania* were emerged only in the wet soil, while *Sulfuricaulis*, *Thermanaerothrix*, *Thermodesulfovibrio*, *Desulfobulbus*, and *Thiohalobacter* were detected only in the water samples. Interestingly, the relative abundance of *Alkaliphilus* in influent was as high as 20.67%, while the sum of all dominant genera in the dry soil was 15.12%.

### 3.3. Archaeal Community Structures in Constructed Wetlands

#### 3.3.1. Archaeal Alpha Diversity Analysis

**Figure 5:**
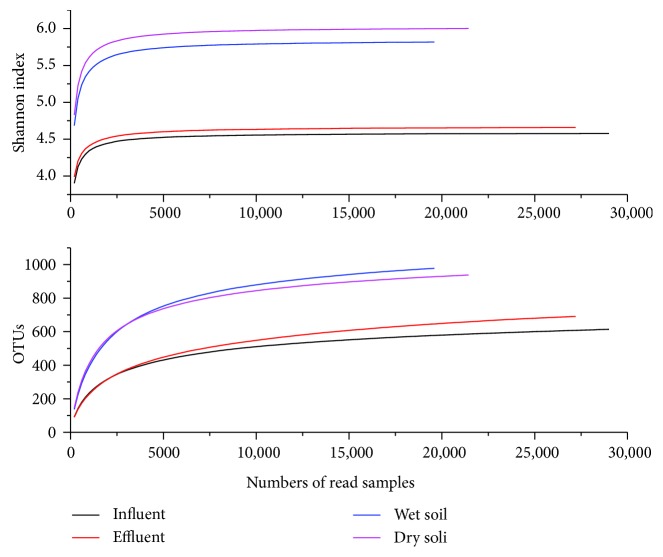
Archaeal rarefaction curves and Shannon diversity index curves.

Rarefaction curves of the four samples were shown in the Figure 5. The rarefaction curves and Shannon diversity index curves of four samples clearly revealed that the archaeal community structures of soil samples were considerably higher than those of water samples. Two kinds of curves tended to be gentle, suggesting that the sequencing results had been enough to reflect the diversity of the current sample, and increasing the depth of sequencing could not detect more new OUTs. The sequencing results could basically reflect the microbial community structures of four samples. The trend changes of rarefaction curves and Shannon diversity index curve between archaea and bacteria were exactly the same.

A total of 56,140, 32,879, 61,599, and 28,301 trimmed reads for samples influent, effluent, wet soil, and dry soil were obtained, respectively, after the removal of unqualified reads (Table 4). The community richness in wet soil sample was much higher than that in the other three samples, suggesting that archaea became active under wet anoxic conditions [17]. The community diversity of archaea in wet soil sample was the highest in this study, while that in effluent sample was the lowest. The Shannon index was more sensitive to the abundance of the community and the rare OTUs, indicating that there were more unidentified species in the archaeal community. In addition to the ACE estimator and Chao1 estimator in effluent and dry soil, the other alpha diversity indices were all higher than the bacterial community structures.

#### 3.3.2. Archaeal Community Structures of Soil and Water in Constructed Wetlands

**Figure 6:**
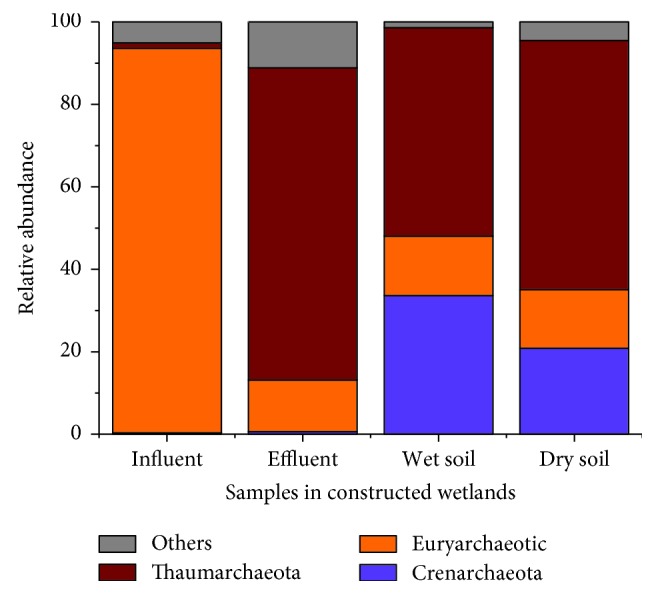
Archaeal relative abundance of four samples in phyla in constructed wetlands.

Archaeal sequences in the four samples were classified into taxonomic classes using the default settings of the Qiime platform. Unlike bacteria, the result of archaea is quite simple and the number of phyla was very low. A total of 3 archaeal phyla were found existing in all four samples, but quite different in compositions, and the detailed relative abundances were shown in Figure 6. Euryarchaeotic were the absolute dominant phylum in the influent sample with the relative abundance of 93.29%, while it was no more than 15% in the other three samples. Thaumarchaeota showed dominance in the other three samples (50.58%~75.70%) but accounted for only 1.28% in the influent. Crenarchaeota were one of the common archaeal phyla in the soil samples with a high relative abundance (20.86% and 33.61%), while the proportion was very low in the water samples (0.34% and 0.61%). Simultaneously, some archaeal phyla and no blast hit sequences were also found in the samples which were classified into others. The community structures of the two soil samples were more similar, while the structures of two water samples were quite different.

**Figure 7:**
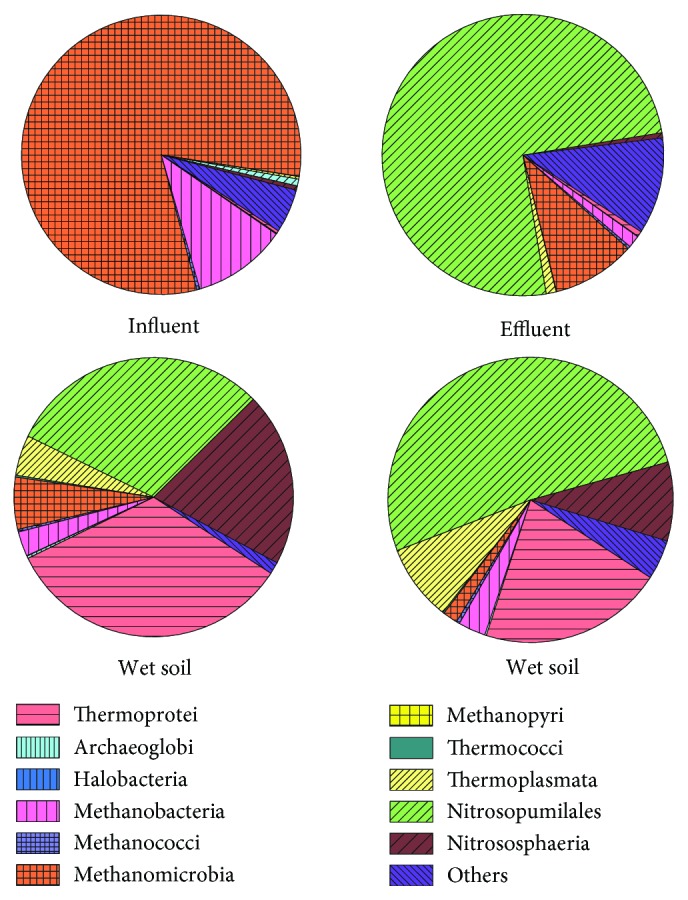
Archaeal relative abundance of four samples in classes in constructed wetlands.

The distribution characteristics of classes were shown in Figure 7. A total of 11 archaeal classes were found, and the total class numbers in influent, effluent, wet soil, and dry soil all were 10. The relative abundances of different classes were quite different. Methanomicrobia and Thermoprotei were the dominant classes in the influent and wet soil, with the relative abundances of 81.58% and 33.61%, while Nitrosopumilales showed advantage in the effluent and dry soil, accounting for 75.12% and 51.51%. It was worth pointing out that the relative abundances of Nitrosopumilales were high not only in the soil samples but also in the effluent (30.44%), while it was very low in the influent, indicating that Nitrosopumilales was nonexistent in the sewage and mainly existed in the wetland matrix. Methanomicrobia had a low concentration in the other three samples, except in the influent, guessing the main source of it was the sewage treatment process. The relative abundances of Thermoprotei, Thermoplasmata, and Nitrososphaeria in soil samples were much higher than those in water samples.

Due to the huge amount of data, the dominant genera, with relative abundances over 1%, were listed in Table 5. A total of 13 archaeal genera were found in this study. The archaeal dominant genera in four samples accounted for 86.83%~95.95% in archaeal microbial communities. In influent sample, *Methanosaeta* and *Methanocorpusculum* were the dominant genera, but they had very low relative abundances in the other three samples. Except for them, the other 11 dominant genera were all less than 7%. The relative abundances of *Nitrososphaera*, *Ignisphaera*, *Staphylothermus*, *Thermodiscus*, and *Methanomassiliicoccus* in soil samples were much higher than those in water samples. It was worth pointing out that *Nitrosopumilus* all had very high relative abundances in effluent, wet soil, and dry soil samples, but less than 1% in influent sample.

## 4. Discussion

### 4.1. Bacterial Diversity and Community of Soil and Water in Constructed Wetlands

To date, little is known about bacterial community structures in the free water surface constructed wetlands. Proteobacteria was the dominant bacteria of all the water and soil samples, with the relative abundance all over the 35% in phyla. The same conclusions had been confirmed in previous coastal water [18], airborne [19], and soil [20] studies, which could prove that Proteobacteria were the dominant phylum in almost all environmental samples. Microorganisms in Proteobacteria were gram-negative bacteria, and a large number of nitrogen-related microorganisms were distributed in Proteobacteria [21, 22]; these may explain why the relative abundances of Proteobacteria in constructed wetlands were higher than those in natural wetlands [23–25]. Bacteroidetes and Firmicutes both belonged to the gut microorganisms [26, 27], and the high relative abundances in this study may be due to the relatively open characteristics of the constructed wetlands, and there were a large number of birds and insects inhabiting the surrounding area, at the same time, Firmicutes were able to degrade a variety of organic pollutants [28], and the sewage treatment systems were their main source, which may explain why the relative abundances of Firmicutes in influent were higher than those in other three samples. Chloroflexi was proved to be a common phylum in various wastewaters from constructed wetland systems [29, 30]; however, in this study, the relative abundances of Chloroflexi in soil were significantly higher than those in water, so this was speculated that some microorganisms would be enriched in the soil, perhaps the same conclusions could be also summarized in Acidobacteria, Ignavibacteriae, Gemmatimonadetes, and Nitrospirae. Currently, numerous studies had found that toxigenic Cyanobacteria [31, 32] and the high content of Cyanobacteria in the effluent should cause the attention of the monitoring department. In this study, three bacterial phyla were emerged only in the water, while four existed only in the soil, indicating that even if the sampling locations were similar, different environmental sample sources would still cause different bacterial community structures.

The dominant bacteria found in study were basically consistent with previous studies. A small amount of *Steroidobacter*, a microcystin-degrading Gammaproteobacterium isolated from soil [33], was found in influent in this study, guessing it might come from the sewage treatment process. *Taibaiella* was the dominant genera in the biofilms [34] and soil [35]; interestingly, it was not found in influent. *Sulfuricaulis* was mainly isolated from sediment of a lake in Japan [36]; however, it was only exited in soil in this study. *Desulfobulbus* was isolated from marine sediment [37] and also only in soil, fit in with the characteristics of the sampling location geographical environment, adjacent to the ocean. In recent years, there had been very little related research on *Alkaliphilus*, but its abundance in influent was as high as 20.67%, which needed our more attentions. *Limnohabitans*, novel planktonic Betaproteobacteria, isolated from a freshwater reservoir, could prove that the quality of water across constructed wetlands had improved significantly. *Calothrix* is the dominant flora in natural water [38], and the relative abundances increased significantly after purification.

This study found that the soil community diversities were lower than those of the water, while the dry soil bacterial structures were simpler than wet soil. *Mycobacterium* is an important global threat to individuals with cystic fibrosis [39], and the relative abundances in water were much higher than those in soil in this study, reduced substantially through the water treatment, which had been confirmed to be correlated with turbidity [40]. *Enterococcus* showed high cholesterol removal ability [41] and were capable of hydrogen production [42], which could lay the foundation for researches on new energy. *Dysgonomonas* could cause liver abscesses [43] and played a major role in the mechanism for electricity generation [44], which were found only in water. *Flavobacterium* caused devastating mortality in various freshwater fish species globally [45] and were isolated from the China No. 1 glacier, as a kind of psychrophilic bacteria [46]. *Pseudomonas* was responsible for chronic infection [47] and was the most common bacteria in the soil [48], and this may explain why the relative abundances of *Pseudomonas* in dry soil sample were higher than those in water and wet soil samples. *Janthinobacterium* may cause a soft rot disease of Agaricus bisporus [49] and were isolated from both water [50] and soil [51]; however, there were no such genus in the soil samples of this study.

### 4.2. Archaeal Diversity and Community of Soil and Water in Constructed Wetlands

The distributions of archaeal abundance in water and soil in constructed wetlands were poorly understood, which increased difficulty in the analysis of this study. Among them, 1.03%~9.07% of the sequences could not find its chimera, and 0.14%~2.07% was identified as bacteria. Three archaeal phyla was found in this study, but the gap between water and soil was very large. Euryarchaeotic, accounting for 93.29%, were the dominant phylum in influent, which is involved in methane production [52]. At present, in the constructed wetland system, there were only a few related researches which revealed that Euryarchaeotic was an advantage phylum [53, 54]. In this study, it had reached as much as 93.29%, which should arouse our attention. Most previous studies suggested that Euryarchaeotic was a major archaeal group in constructed wetland system [55, 56], but the influential factors, which affect the relative abundance of Euryarchaeotic, were not yet clear [57]. Thaumarchaeota was a marine archaea and abundant ammonia-oxidizers [58], which ensured the purification efficiency of constructed wetlands and had been widely reported before [59, 60]. This study also found a small amount of Crenarchaeota, which had a high abundance in a temperate acidic forest soil [61], and this conclusion was also consistent with the water quality of the constructed wetland.

Previous researches had reported *Nitrososphaera* and *Nitrosopumilus* [62, 63] belonged to ammonia-oxidation archaea, their large amount of existence could guarantee the purification effect of the constructed wetland system. The relative abundances of *Nitrosopumilus* increased suddenly after passing through the constructed wetlands, from 0.79% to 75.12%, which may be mainly related to the concentration of dissolved oxygen. *Methanomassiliicoccus*, *Methanosarcina*, *Methanomethylovorans*, *Methanocorpusculum*, *Methanobrevibacter*, and *Methanobacterium* were all classified as methanogenic archaea had great potentials for different industrial uses [64]. The methanogenic archaeon *Methanomassiliicoccus* was isolated from human feces [65], and the discovery of it filled the blank of the natural coal-based methanogen group records. *Methanosarcina* played an important role in the long-term bioremediation of uranium-contaminated aquifers and had the potential to influence uranium geochemistry in a diversity of anaerobic sedimentary environments [66]. *Methanosaeta* had only been reported once in the past three years [67], and its research should be strengthened later. *Methanomethylovorans* was also a methylotrophic archaea and had a great potential as additional inoculum for bioreactors to carry out biogas production and other related processes [68]. *Desulfurococcus* was an anaerobic, hyperthermophilic crenarchaeon and able to use a variety of different carbon sources [69]. In addition to the genera mentioned above, this study could not find the previous studies on *Thermodiscus*, *Staphylothermus*, and *Ignisphaera*, which should be emphasized in later studies because of their high relative abundances in soil samples.

## 5. Conclusion

Taken together, the present study, using the Illumina MiSeq high-throughput sequencing method, provided a detailed picture of bacterial and archaeal community variations on phylum, classes, and genus level under the full-scale constructed wetlands. Sequencing results and alpha diversity indices indicated that the total bacterial OTUs could be assigned into 29 different phyla, while archaeal OTUs were only 3. Among them, Proteobacteria were the most dominant bacterial phyla with the relative abundance of 35.38%~48.66%. Euryarchaeotic and Thaumarchaeota were the dominant archaeal phyla. The diversity of bacterial community structure was significantly higher than that of archaea simultaneously, and the community structures of soil microorganisms were obviously different from the water microorganisms. At genus level, nine bacterial genera had close relation with animal or plant diseases, which could be used for microbial risk assessment simultaneously, and archaeal genera were mainly concentrated in methanogens or anaerobic archaea, which might provide some useful microbial information for the bioremediation. It is worth noting that the lack of researches in archaea had brought great difficulties to this study, which should be emphasized in later studies.

## Figures and Tables

**Table 1:** Physical and chemical characteristics in samples.

Samples	Total phosphorus (g/kg)	Total nitrogen (g/kg)	Organic matter (g/kg)	Samples	Dissolved oxygen (mg/L)	Ammonia nitrogen (mg/L)	Nitrite nitrogen (mg/L)	pH
Dry soil	2.66	22.12	391.61	Influent	8.93	6.05	1.06	6.97
Wet soil	0.38	7.56	26.75	Effluent	11.53	1.30	0.36	6.95

**Table 2:** Bacterial alpha diversity indices of four samples.

Samples	Reads	OTUs	ACE	Chao1	Shannon	Simpson
Influent	29,000	614	676.31	707.02	4.58	0.05
Effluent	27,204	691	792.39	783.76	4.66	0.03
Wet soil	19,597	978	1051.89	1080.43	5.82	0.01
Dry soil	21,439	938	1002.35	1019.01	6.00	0.01

**Table 3:** The bacterial dominant genera in four samples in constructed wetlands.

Name of similar genera	Influent (%)	Effluent (%)	Wet soil (%)	Dry soil (%)
*Aliterella*	—	1.39	0.01	—
*Alkaliphilus*	20.67	0.36	0.01	—
*Arcobacter*	4.74	0.24	0.34	—
*Bacillus*	0.12	1.46	1.60	1.69
*Calothrix*	0.71	12.92	0.01	0.49
*Curvibacter*	1.33	0.21	0.01	0.01
*Dechloromonas*	3.03	0.13	0.29	0.05
*Desulfobulbus*	—	—	1.05	0.07
*Dysgonomonas*	1.08	0.05	—	—
*Enterococcus*	0.30	1.61	1.58	1.48
*Flavobacterium*	0.09	10.93	0.13	0.54
*Fluviicola*	0.29	1.53	0.04	—
*Gemmatimonas*	0.12	0.01	1.10	2.41
*Gemmobacter*	0.10	1.18	0.03	—
*Haliscomenobacter*	—	2.40	—	—
*Herminiimonas*	4.82	0.33	—	—
*Hydrogenophaga*	0.21	2.58	0.18	0.04
*Janthinobacterium*	0.17	1.85	—	—
*Lactococcus*	0.23	2.26	3.22	3.11
*Limnohabitans*	0.07	6.83	—	—
*Mycobacterium*	8.33	1.35	0.10	0.11
*Nordella*	1.09	0.21	0.04	0.03
*Owenweeksia*	—	1.20	—	—
*Polaribacter*	—	1.30	—	—
*Prevotella*	1.56	0.15	—	—
*Pseudomonas*	0.22	0.08	0.32	1.40
*Rhodoferax*	2.97	4.08	0.96	0.08
*Sediminibacterium*	0.33	1.03	—	—
*Steroidobacter*	0.03	—	1.06	0.36
*Sulfuricaulis*	—	—	0.61	2.00
*Synechococcus*	—	1.60	—	—
*Tabrizicola*	0.07	1.03	0.09	0.01
*Taibaiella*	—	1.21	0.02	0.04
*Tangfeifania*	—	—	6.71	—
*Thermanaerothrix*	—	—	0.01	1.07
*Thermodesulfovibrio*	—	—	1.19	0.50
*Thiobacillus*	0.06	0.01	3.61	1.96
*Thiohalobacter*	—	—	1.35	0.03
*Trichococcus*	1.10	0.03	—	—
*Vogesella*	1.12	0.03	—	—

**Table 4:** Archaeal alpha diversity indices of four samples.

Samples	Reads	OTUs	ACE	Chao1	Shannon	Simpson
Influent	56,140	2185	850.94	817.04	6.23	0.96
Effluent	32,879	1752	727.75	714.25	5.61	0.92
Wet soil	61,599	3994	1510.08	1454.2	7.54	0.98
Dry soil	28,301	1546	773.00	773.00	6.49	0.97

**Table 5:** The archaeal dominant genera in four samples in constructed wetlands.

Name of similar genera	Influent (%)	Effluent (%)	Wet soil (%)	Dry soil (%)
*Nitrososphaera*	0.49	0.57	20.14	8.93
*Nitrosopumilus*	0.79	75.12	30.44	51.51
*Methanomassiliicoccus*	0.24	0.36	4.48	8.61
*Methanosarcina*	2.39	0.02	2.68	0.37
*Methanomethylovorans*	1.13	0.33	0.03	—
*Methanosaeta*	42.44	3.04	2.16	0.14
*Methanocorpusculum*	34.42	5.60	0.12	—
*Methanobrevibacter*	6.29	0.75	0.44	0.45
*Methanobacterium*	3.95	0.41	1.89	2.53
*Thermodiscus*	0.24	0.50	25.53	14.08
*Staphylothermus*	0.06	0.05	1.73	1.89
*Ignisphaera*	0.02	0.04	4.58	3.17
*Desulfurococcus*	0.02	0.02	1.77	1.71

## Data Availability

The data used to support the findings of this study are available from the corresponding author upon request.
